# Patterns of Cannabidiol (CBD) Use and Mental Health Correlates in Young Adults: A Cross-Sectional Study

**DOI:** 10.1192/j.eurpsy.2026.10850

**Published:** 2026-07-31

**Authors:** O. Martin-Santiago, B. Arribas-Simon, M. Calvo-Valcarcel, P. Martinez-Gimeno, M. Rios-Vaquero

**Affiliations:** Hospital Clinico Universitario de Valladolid, Valladolid, Spain

## Abstract

**Introduction:**

Cannabidiol (CBD), a non-psychoactive compound derived from *Cannabis sativa*, has gained popularity among young adults seeking natural alternatives for managing emotional and physical symptoms. Despite its increasing use, particularly without medical supervision, scientific evidence regarding its safety and efficacy remains limited.

**Objectives:**

This study aimed to:

- Describe the demographic and clinical profile of CBD users.
- Assess perceived effects of CBD on mental health and well-being.
- Explore associations between CBD use, mental health history, and cannabis consumption.

**Methods:**

A cross-sectional survey was conducted among 230 individuals aged 18–40, recruited via social media. Data collected included sociodemographic variables, mental health history, substance use, and perceptions of CBD efficacy.

**Results:**

Among the participants, 14.3% reported having used CBD. These individuals showed a higher prevalence of mental health care, depression, antidepressant use, and prior cannabis consumption compared to non-users (*see* ***Table 1* *</b>***). The most common motivations for CBD use were stress/anxiety relief and peer recommendations. Notably, a large majority (81.8%) initiated CBD use without prior consultation with a healthcare professional.

**Table 1.** Mental Health and Substance Use Characteristics of CBD Users vs. Non-Users Table 1. Long description.

Variable	CBD Users (%)	Non-Users (%)
Mental health care	57.6	41.6
History of depression	30.3	15.7
Antidepressant use	24.2	10.2
Previous cannabis use	39.4	8.1
Never used cannabis	18.2	53.3

Perceived efficacy varied by user profile. As shown in **Image 1**, 75% of cannabis users reported emotional improvement with CBD, while 73.3% of non-cannabis users did not perceive any benefit (*p* = .026). Similarly, participants with a history of depression were more likely to rate CBD as “very effective” (75%) compared to those without such history (25%), while 88.9% of those who found CBD ineffective had no depression history (*p* = .048).

**Image 1:**
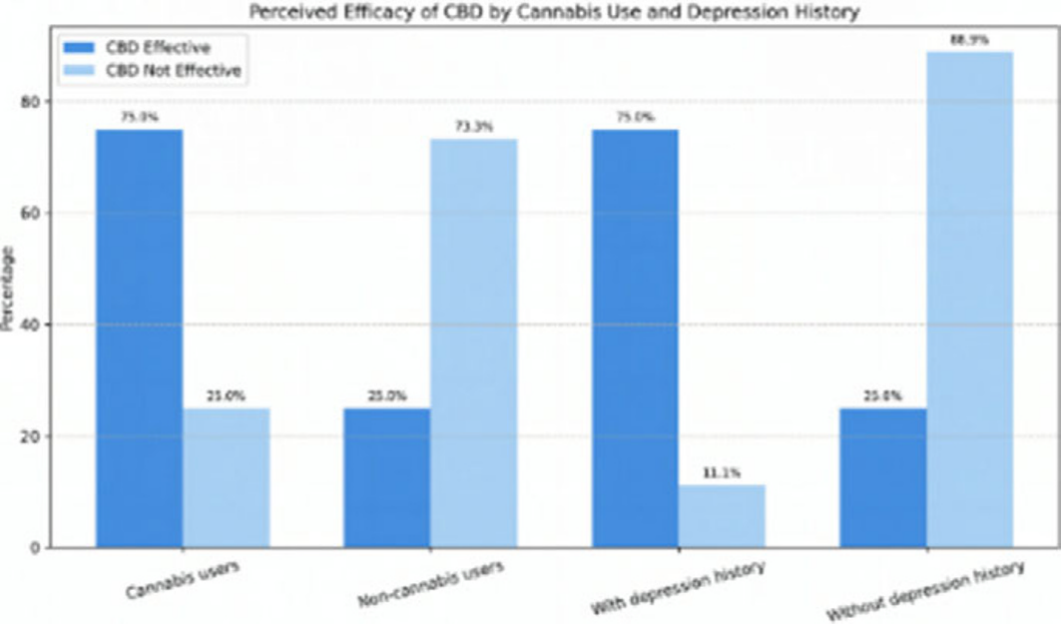
Image 1: Long description.

**Conclusions:**

This study reveals a strong association between CBD use and mental health vulnerability, particularly among individuals with depressive symptoms or prior cannabis use. Compared to previous studies that emphasized insomnia or chronic pain as primary motivations, our findings highlight stress and anxiety relief as dominant factors. The high rate of unsupervised consumption underscores the need for regulatory oversight and public health education. These results align with prior research indicating increased self-medication behaviors among emotionally vulnerable populations and suggest that CBD may be perceived as a therapeutic alternative, especially by those already engaged in psychopharmacological treatment.

**Disclosure of Interest:**

None Declared

## Long descriptions

### Table 1. Long description

The table consists of three columns: Variable, C B D Users (percent), and Non-Users (percent).

* Mental health care: 57.6 percent for C B D users versus 41.6 percent for non-users.

* History of depression: 30.3 percent for C B D users versus 15.7 percent for non-users.

* Antidepressant use: 24.2 percent for C B D users versus 10.2 percent for non-users.

* Previous cannabis use: 39.4 percent for C B D users versus 8.1 percent for non-users.

* Never used cannabis: 18.2 percent for C B D users versus 53.3 percent for non-users.

Navigate back to Table 1..

### Image 1: Long description

A grouped bar chart titled Perceived Efficacy of C B D by Cannabis Use and Depression History. The y-axis is labeled Percentage and ranges from 0 to 80 with increments of 20. The x-axis contains four categories. A legend in the top left indicates that dark blue bars represent C B D Effective and light blue bars represent C B D Not Effective.

Data points from left to right are as follows.

* Cannabis users. C B D Effective is 75.0 percent and C B D Not Effective is 25.0 percent.

* Non-cannabis users. C B D Effective is 25.0 percent and C B D Not Effective is 73.3 percent.

* With depression history. C B D Effective is 75.0 percent and C B D Not Effective is 11.1 percent.

* Without depression history. C B D Effective is 25.0 percent and C B D Not Effective is 88.9 percent.

Navigate back to Image 1:.

